# Assessing the Performance of Mass Spectrometry Search Strategies in Identifying Translational Errors Using PDX Proteomics Data

**DOI:** 10.1016/j.mcpro.2025.101500

**Published:** 2025-12-22

**Authors:** Araf Mahmud, Yingnan Song, Qi Zhou, Chen Huang

**Affiliations:** 1 Department of Genetics, University of Alabama at Birmingham, Birmingham, Alabama, USA; 2 O'Neal Comprehensive Cancer Center, University of Alabama at Birmingham, Birmingham, Alabama, USA

**Keywords:** open search, patient-derived xenograft, translational errors, benchmarking, single amino acid variation

## Abstract

Translational errors (TEs) result in a mismatch between mRNA codons and the amino acids (AAs) of the corresponding protein. Unlike DNA mutations or RNA editing, where nucleotide sequences can be used to infer AA substitutions, TEs can only be detected at the protein level. Although high-throughput mass spectrometry (MS) proteomics offers the potential to resolve peptide sequences and could theoretically be used to identify TEs, the feasibility of current MS data analysis approaches for this application remains uncertain. Here, we utilize patient-derived xenograft proteomics data, which include both human and mouse peptides with identifiable cross-species AA variations, as a ground truth for benchmarking TE identification methods. By using high-confidence mouse peptides as surrogates for “TE-containing” peptides, we show that current open search approaches can achieve >65% overall sensitivity and >70% overall precision for high-quality samples. The intersection of different search strategies significantly enhances precision, albeit at the expense of reduced sensitivity. Notably, the evaluation metrics vary significantly across individual AA substitutions, suggesting that caution is warranted when detecting or interpreting specific AA substitutions. Moreover, closed searches targeting predefined AA changes exhibit poor precision, with post-translational modification mislocalization identified as a key bottleneck for this application. Overall, our study provides a first-of-its-kind benchmark for MS-based TE discovery and offers guidance for optimizing MS search strategies.

## Highlights

• PDX proteomics data enable rigorous evaluation of mistranslation search strategies.
• Open searches can reach >70% precision and >65% sensitivity in high-quality samples.
• Identification performance varies across different amino acid substitutions.
• Closed searches are limited by PTM mislocalization for translational error IDs.

## Graphical Abstract

**Figure:**
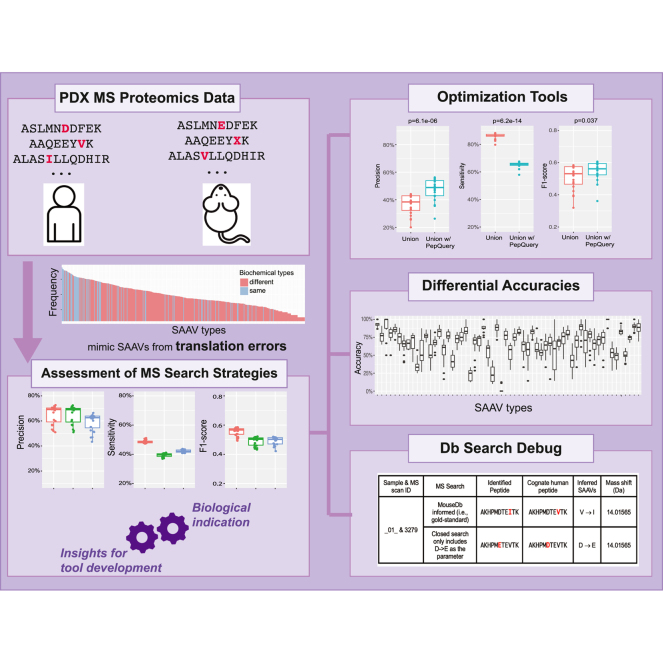

## In Brief

Amino acid substitutions caused by translational errors have been explored through the secondary analysis of large-scale mass spectrometry proteomics data. However, the effectiveness of current proteomics data analysis methods in identifying these substitutions has not been thoroughly assessed in this context. Here, we demonstrate that cross-species single amino acid variants in patient-derived xenograft proteomics data provide a robust framework for this assessment. We benchmarked open and closed search strategies at the peptide-spectrum match level, offering insights for biological discovery and tool optimization in translational error-single amino acid variant identification.

Proteins, as the final products of the central dogma, can have their sequences altered by abnormalities at various levels. In complex diseases like cancer, somatic or germline mutations at the DNA level and RNA editing are well-recognized sources for modifying protein sequences (1). In recent years, proteogenomics, leveraging next-generation sequencing and mass spectrometry (MS), has provided a powerful framework for identifying abnormalities at the DNA level or the RNA level that can alter protein sequences (2). Specifically, proteogenomics uses disease- or sample-specific next-generation sequencing data to infer peptide candidates that are absent from standard protein databases (*e.g.*, UniProt or RefSeq), followed by MS spectrum searches to examine their existence at the protein level (3). There are well-established tools for protein database customization and benchmarking studies to evaluate their reliability (4, 5, 6).

However, protein sequence alteration driven by translational errors (TEs) remains underexplored (7). TEs, also known as translational infidelity, result in a mismatch between the codons of an mRNA and the actual amino acids (AAs) incorporated into the corresponding protein. For example, TEs can be caused by tRNA mischarging, where an AA is mistakenly attached to a tRNA that normally carries a different AA (8). Although earlier studies suggest that the TE rate is low under normal physiological conditions (9), increasing evidence suggests that TEs might be more common under cell stress or specific disease conditions (10), such as AA depletion (11). In certain diseases, such as cancer, TEs have drawn increasing attention as peptides containing AA substitution might serve as therapeutic targets (12).

Recently, several studies have explored TE-derived single amino acid variations (TE-SAAVs) from large-scale MS proteomics data, such as those generated by the Clinical Proteomic Tumor Analysis Consortium (CPTAC). Mordret *et al*. (13) and Tsour *et al*. (14) employed a MaxQuant-dependent search, which assumes that both the peptide with TE SAAVs and its cognate base peptide (*i.e.*, genome encoded) exist in the proteomic data, as TEs usually occur in only a proportion of peptide copies. In comparison, Pataskar *et al*. (11) used a customized search database that includes potential peptides with AA substitutions. Although these findings are intriguing, none of the involved approaches has been systematically tested against a robust ground truth. The challenge in benchmarking TE-SAAV search strategies lies in the lack of ground truth, as TEs cannot be inferred by DNA or RNA sequence evidence. Even if one were to use the SAAVs from somatic mutations as a proxy for TE-associated SAAVs for benchmarking purposes, the limited number of protein-coding mutations cannot provide a comprehensive SAAV-containing peptide pool to mimic TEs, which may occur broadly across the whole proteome. Moreover, it is unknown whether the SAAV patterns resulting from somatic mutation are similar to those caused by TEs.

Patient-derived xenografts (PDXs) are experimental models where tumor tissue from human patients is transplanted into immunocompromised mice (15). Due to mouse cell infiltration (primarily stromal and myeloid immune cells), PDX samples contain both human and mouse tissues. Given the numerous mouse–human homologous protein pairs with differential AA usages, PDX proteomics data offer a valuable source for evaluating TE discovery strategies by treating mouse peptides containing cross-species SAAVs as surrogates for TE-SAAVs. Beyond providing more SAAVs per sample for benchmarking, PDX proteomics data have two key advantages. First, the well-defined mouse sequence enhances confidence in establishing the ground truth. Second, cross-species AA discrepancies might provide an SAAV substitution pattern that better reflects TE-SAAVs.

In this study, we used recently published high-resolution PDX MS proteomics data to assess the sensitivity and precision of different MS search strategies for TE-SAAV discovery. We focused particularly on the open search strategy, which does not rely on predefined SAAVs and can be applied broadly to proteomics data from biological samples in diverse disease conditions.

## Experimental Procedures

### Tools and Data Sources

MaxQuant (version 2.4.2.0), Open-pFind (version 3.2.0), and MSFragger (version 4.1, run within FragPipe 22.0) were downloaded from their official websites.

The two PDX proteomics datasets were downloaded from PRIDE (https://www.ebi.ac.uk/pride/). The pancreatic cancer PDX dataset (PXD042267) comprises 48 samples, of which one duplicate set (24 samples) was selected for use, whereas the prostate cancer PDX dataset (PXD009636) includes 17 samples, all of which were utilized. The .raw files were processed using MaxQuant and Open-pFind, whereas .mzML files, converted from .raw files using MSConvert (version 3.0.23), were used for MSFragger.

AutoRT (version 1.0, https://github.com/bzhanglab/AutoRT), pDeepQuery (version 1.0, https://github.com/pFindStudio/pDeep/tree/master/pDeep2), and PepQuery (version 2.0.2, http://www.pepquery.org) were downloaded from their respective websites.

The Cancer Genome Atlas (TCGA) somatic mutation data for 33 cancer types were downloaded using TCGAbiolink (2.31.4) (16) and were analyzed to determine the number of SAAV types resulting from missense mutation.

### MS Data Search

#### MouseDb-Informed Search

To establish the gold-standard data, a concatenated mouse and human National Center for Biotechnology Information RefSeq protein reference database was employed for closed searches conducted with FragPipe and MaxQuant (17). The RefSeq human and mouse reference proteomes were downloaded on March 28, 2023, and processed into the FASTA data format. Entries were annotated with the National Center for Biotechnology Information GeneID, TaxonID, HomologeneID, and gene symbol. The reference database contains 80,872 human protein sequences and 62,232 mouse protein sequences.

The following parameters were applied: 20 ppm for precursor tolerance; 20 ppm for fragment mass tolerance; trypsin (no cleavage after proline, allowing up to two missed cleavages) as the enzyme; cysteine carbamidomethylation as a fixed modification; methionine oxidation and protein N-terminal acetylation as variable modifications; and 6 to 61 AAs as the range of peptide length. The false discovery rates of identification, based on the target-decoy search strategy, were set to the default (0.01) for both tools at peptide-spectrum match (PSM) and peptide levels.

### Open Search

For evaluating the performance of open search, only human proteins were used as the reference. MSFragger open search (*i.e.*, “MSFragger-open”), MaxQuant-dependent search (*i.e.*, “MaxQuant-dp”), and Open-pFind utilized default open search parameters. Open-pFind was set to “HCD FTMS” mode. For MaxQuant-dp, “dependentPeptides” was set to True. Other parameters, including fixed and variable post-translational modifications (PTMs), PSM-level, and peptide-level false discovery rates, and peptide length range, were consistent with the closed search settings.

### Closed Search With Preset SAAVs as PTM Parameters

This closed search was conducted similarly to the MouseDb-informed search, with the following exceptions: (1) only the human protein reference database was provided. (2) One of the selected SAAVs (*e.g.*, D→E, V→X, or I→V) was added to the variable modifications.

### Closed Search With Customized Protein Database

MAF files containing somatic mutation results for TCGA and CPTAC pancreatic cancer and prostate cancer cohorts were downloaded from the Firehose (https://gdac.broadinstitute.org/) and CPTAC Pan-Cancer (https://proteomic.datacommons.cancer.gov/pdc/cptac-pancancer) data portals, respectively. Proteins containing missense mutations and indels were added to the human and mouse concatenated database for the MouseDb-informed search corresponding to the cancer type. In addition, RNA-Seq raw data for the prostate PDX samples (https://www.ebi.ac.uk/ena/browser/view/PRJEB9660) (18) were downloaded to derive sample-specific somatic mutations using the nf-core pipeline “rnadnavar” (https://nf-co.re/rnadnavar/dev/). The resulting missense and indel mutations were similarly included for the customized database search.

### Collection of Gold-Standard PSMs

The ground truth consists of PSMs corresponding to the mouse peptides that contain an SAAV relative to their cognate human peptides and were also expressed based on the PDX proteomics datasets (*i.e.*, identifiable by the proteomics). First, all human and mouse cognate gene symbols were retrieved from the Jackson Laboratory (https://www.informatics.jax.org/genes.shtml) and used to obtain protein sequences from the GENCODE human (release 47, https://www.gencodegenes.org/human) and mouse (release M36, https://www.gencodegenes.org/mouse) protein databases. Subsequently, *in silico* protein digestion was performed using the same parameters as the MS search. All mouse and human peptide pairs with an SAAV were retained, representing the theoretical cognate mouse and human peptides.

Next, all peptides identified from the MouseDb-informed search by MSFragger or MaxQuant were intersected with the theoretical cognate mouse and human peptides. The PSMs with overlapping mouse peptides served as the gold standard for the MSFragger open search, Open-pFind open search, and closed search with preset PTMs. For the MaxQuant-dependent peptide search, the gold-standard peptides were obtained by further filtering the overlapping mouse peptides to include only those whose cognate human peptides were identified in the same samples.

### Parsing of Open Search Results

The SAAVs from the Open-pFind open search are included directly in the search results. In contrast, results from the MSFragger open search and MaxQuant-dependent peptide search provide only the mass difference between the experimental spectra and the baseline peptide sequence, along with possible PTM locations within the peptide. To align with the gold-standard PSMs, we performed PSM filtering following the open search. First, we selected PSMs with a confidently assigned PTM location from the MSFragger open search and those with a single location probability greater than 0.5 from the MaxQuant-dependent peptide search. Next, a PTM was classified as an SAAV if the following criteria were all met: (1) the absolute difference between the mass shift caused by the SAAV and the mass shift induced by the PTM (*i.e.*, Δmass shift) is the smallest among all theoretical AA pairs, (2) the Δmass shift is less than 0.1 Da, and (3) the starting AA in the SAAV matches the AA in the baseline sequence at the identified location. PSMs that did not satisfy all three criteria could not be assigned an SAAV and were excluded from downstream analysis.

### Evaluation of Open Search Results at the PSM Level

The evaluation was conducted at the PSM level. Specifically, when the MouseDb-informed search mapped a mouse peptide to a spectrum (*i.e.*, generating a PSM), the evaluation assessed whether the open search could achieve the same mapping. To this end, we collected all gold-standard PSMs from the MouseDb-informed search, as described above. The PSMs from the open search or targeted PTM search, which utilized the human protein database, were parsed and filtered to identify those corresponding to peptides with SAAVs to obtain candidate PSMs. True positives were defined as the candidate PSMs that overlapped with the gold-standard PSMs. Sensitivity and precision were calculated using the following formulas:

$$\text{Sensitivity}=\frac{\#\;\text{True}\;\text{postive}\;\text{PSMs}}{\#\;\text{Total}\;\text{gold}-\text{standard}\;\text{PSMs}}\times 100\%$$

$$\text{Precision}=\frac{\#\;\text{True}\;\text{postive}\;\text{PSMs}}{\#\;\text{Total}\;\text{candidate}\;\text{PSMs}}\times 100\%$$

Since the MouseDb-informed search results generated by MaxQuant and MSFragger were not fully consistent, and recognizing that neither of them is perfect, we established a more tolerant ground truth in addition to using their results separately. When assessing sensitivity, the intersection of results from both tools was used to derive the true positives and as the denominator, ensuring that only high-quality ground truth was used for recall. Conversely, the union of results was used to assess precision, minimizing the exclusion of true PSMs from candidates. We designated the intersected or unioned PSMs as the “mixed gold-standard PSMs,” which constituted the tolerant ground truth.

In addition, we used the F1 score as an evaluation metric that balances sensitivity and precision. The F1 score is calculated as follows:

$$F1=2\cdot \frac{\text{Precision}\cdot \text{Sensitivity}}{\text{Precison}+\text{Sensitivity}}$$

### PSM Evaluation by Third-Party Tools

AutoRT, pDeep2, and PepQuery were used to evaluate the open search results according to their respective instructions. For AutoRT, the pretrained deep neural network models were used as the base models for transfer learning in this study. Specifically, the base models were fine-tuned into run-specific models using high-quality peptides that were both identified from MSFragger and MaxQuant MouseDb-informed MS search. The run-specific models were used to predict retention times (RTs) for all PSMs. The difference between observed RT and predicted RT, denoted as RT_delta_, was calculated for each PSM as follows:

$${\text{RT}}_{\text{delta}}=\left|\;{\text{RT}}_{\text{observed}}\;-\;{\text{RT}}_{\text{predicted}}\;\right|$$

A smaller RT_delta_ value indicates higher PSM quality.

For pDeep2, the pretrained model was used to predict the MS/MS spectrum based on the peptide sequence. The normalized spectral angle (SA) between the predicted spectrum and the experimental spectrum was calculated as previously described (19):

$$\text{SA}=1-\frac{2\;\cos^{-1}\left(S_{1}-S_{2}\right)x^{2}}{\pi }$$

where S_1_ and S_2_ are the normalized spectra for the experimental and predicted spectrum, respectively. A greater SA value indicates higher PSM quality.

PepQuery analysis was performed using the PepQuery standalone version. All the peptides identified from the open search were individually tested. The PSMs with “Yes” in the “confident” column were positive identifications by PepQuery.

## Results

### A Survey of Mouse-Human Cross-Species SAAVs

**Fig. 1:**
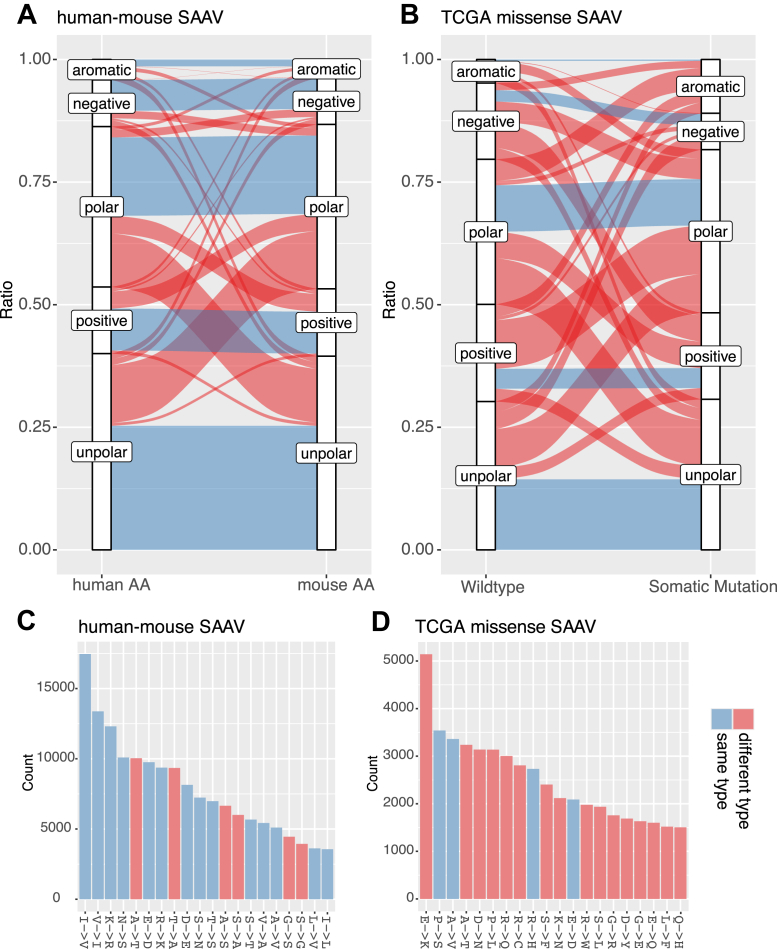
**Comparison of human–mouse cross-species SAAVs and somatic mutation–related SAAVs.***A* and *B,* alluvial diagrams illustrating the ratios of amino acid (AA) substitution categories for human–mouse cross-species SAAVs (*A*) and somatic mutation–related SAAVs (*B*). *Blue flow bands* represent AA substitutions within the same biochemical type, and *red flow bands* indicate AA substitutions across different AA biochemical types. *C,* bar plot displaying the number of peptides containing the top 20 most frequent human–house cross-species SAAVs. *D,* bar plot showing the number of the top 20 most frequent somatic mutation–related SAAVs in TCGA samples. The color scheme is the same as in *A* and *B*. SAAV, single amino acid variation; TCGA, The Cancer Genome Atlas.

Previous studies have shown that TEs caused by tRNA mischarging and codon–anticodon mismatches most frequently generate SAAVs with similar biochemical properties, such as Val/Ile (nonpolar) (20), Met/Leu (nonpolar) (21, 22), and Tyr/Phe (aromatic) (23, 24). Because SAAVs resulting from molecular evolution are less deleterious, we hypothesized that a mouse–human mixed protein pool would serve as a valuable resource for assessing the performance of MS search tools in identifying TE-related SAAVs. We compiled a list of all potential mouse–human SAAVs that might exist in PDX samples. In parallel, we also assembled and cataloged SAAVs caused by missense somatic mutations across TCGA cancer samples, which have commonly been used to benchmark SAAV identification algorithms (25). Overall, cross-species SAAVs exhibit a stronger bias toward AAs within the same biochemical categories compared with those caused by missense mutations (57.5% *versus* 30.4%, *p* < 0.001, Fisher’s exact test) (Fig. 1*A*). Fourteen of the top 20 cross-species SAAVs belong to the same biochemical category, with the two most frequent being the well-documented Ile→Val and Val→Ile (human→mouse). In contrast, SAAVs resulting from somatic mutations display a significantly different profile, with only four of the top 20 belonging to the same biochemical category (Fig. 1*B*). In addition, we compared the two SAAV groups to the TE-SAAV list identified from a recent pan-cancer study (26). Indeed, TE-SAAVs from the study showed significant enrichment of SAAVs with the same biochemical properties, which were only found in cross-species SAAVs but not mutation-derived SAAVs (Supplemental Fig. S1*A*). Moreover, TE-SAAVs overlap more extensively with cross-species SAAVs (95.7%) than with mutation-derived SAAVs (36.4%) (Supplemental Fig. S1*B*). Therefore, cross-species SAAVs offer greater biological relevance than those caused by somatic mutations for evaluating computational approaches to identifying TE-related SAAVs.

### Collection of Gold-Standard Peptides With SAAVs

**Fig. 2:**
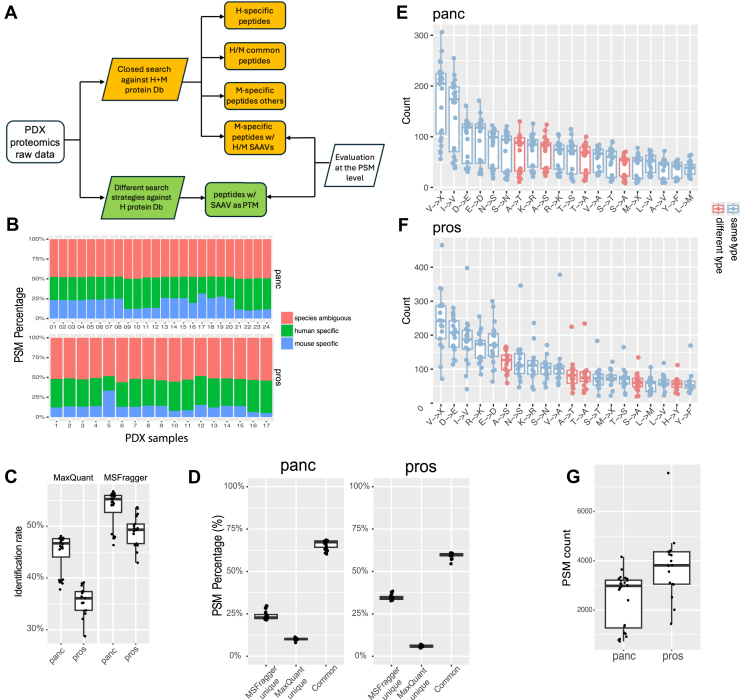
**Workflow and mass spectrometry (MS) data for the study.***A,* schematic diagram of the study design. *Yellow boxes* indicate the ground truth generation, whereas *green boxes* represent the MS search using only human proteins. H, human; M, mouse. *B,* sample-wise composition of human and mouse PSMs from MouseDb-informed MaxQuant search for the two datasets. Colors denote PSMs with different species specificity. *C,* identification rates for the two datasets using MaxQuant and MSFragger. *D,* overlap of PSMs identified by each tool. *E* and *F,* box plots showing the per-sample abundance of the top 20 SAAVs in the pancreatic cancer datasets (*E*) and the prostate cancer datasets (*F*). *G,* box plots showing the per-sample number of mixed gold-standard PSMs in the two datasets. Panc, pancreatic cancer dataset; Pros, prostate cancer dataset; PSM, peptide-spectrum match; SAAV, single amino acid variation.

The overall evaluation is presented in Figure 2*A*. The gold-standard peptides with discoverable “TEs” were defined as the expressed mouse peptides that have SAAVs compared with their cognate human peptides. Unlike previous studies with evaluation at the peptide level (25, 27), our assessment was conducted at the PSM level, offering deeper insights into spectral features potentially associated with SAAV identification. The gold-standard PSMs were identified using a closed search against a concatenated human and mouse protein database (hereafter referred to as the *MouseDb-informed search*) (17, 28). The objective of this benchmarking was to assess how comprehensively and accurately various proteomic data processing approaches can detect these gold-standard PSMs when only the human protein database is provided.

We utilized two recently published PDX proteomic datasets from pancreatic cancer (29) and prostate cancer (30) that were generated by Fourier transform mass spectrometry, which provides high-resolution MS2 spectra for distinguishing SAAVs. MouseDb-informed searches were conducted using MaxQuant (31) and MSFragger (32). MaxQuant search identified 10.0% to 31.4% (median, 30.0%) of all PSMs as mouse specific in the pancreatic cancer dataset and 5.1% to 33.4% (median, 23.2%) in the prostate cancer dataset (Fig. 2*B*). MSFragger’s search results shown nearly identical PSM species composition (Pearson’s correlation >0.99 with MaxQuant results, *p* < 2.2e-16, Supplemental Fig. S1, *C* and *D*). Notably, both datasets exhibited substantial identification rates (median, 55.2% and 49.3% by MSFragger and MaxQuant, respectively), reflecting high data quality (Fig. 2*C*). Among these, the identification rates for the pancreatic cancer dataset were significantly higher, and this dataset was used as the primary evaluation platform, whereas the prostate cancer dataset was used for confirmation purpose.

The mouse PSMs were further filtered to retain only those containing SAAVs in the peptides. As expected, MaxQuant and MSFragger produced significantly overlapping search results for these mouse PSMs (median PSM overlap percentages of 67.2% and 60.0% for the two datasets, respectively). Since there was still a discrepancy between the results from each search tool, we utilized them separately to establish the ground truth (Fig. 2*D*, see Supplemental Note for additional information). Furthermore, we prioritized higher stringency for the gold-standard PSMs when evaluating their recall rate (*i.e.*, sensitivity) and higher inclusiveness when assessing the relevance of candidate peptides (*i.e.*, precision). To this end, we also incorporated the intersection and union of gold-standard PSMs from the two tools to evaluate sensitivity and precision, respectively (hereafter referred to as the *mixed gold standard*). The mixed gold-standard PSMs represented the most lenient ground truth, designed to assess the maximum potential capability for identifying TE-related SAAVs.

Consistent with the global SAAV pattern in Figure 1, most SAAVs from gold-standard PSMs in both datasets share similar biochemical properties (Fig. 2, *E* and *F*). The median per-sample count of gold-standard PSMs was 2980 for the pancreatic cancer dataset and 3814 for the prostate cancer dataset (Fig. 2*G*). Moreover, each sample has, on average, 54 SAAV types with >10 PSMs and 47 SAAV types with >10 unique peptides (Supplemental Note). Compared with evaluations based on mutation-related SAAVs, which typically include fewer than 20 total peptides and 5 SAAV types per sample (25, 27), our gold-standard dataset provided significantly greater power for evaluating SAAV identification by MS data analysis approaches.

In addition, we assessed whether the MS proteomics data from PDX samples contained additional SAAVs caused by somatic mutations, which could affect our search settings and result interpretation. To this end, we built customized protein databases incorporating SAAVs from missense mutations supported by large-cohort cancer studies (*e.g.*, TCGA and CPTAC; see the Experimental procedures section). For the prostate PDX samples, the customized protein database was also constructed by including inferred missense mutations from matched RNA-Seq data. These customized searches did not identify any mutation-derived SAAVs. This finding is not surprising, as CPTAC studies have shown that mutation-derived SAAVs are rarely detected in MS proteomics, ranging from approximately 10 per sample in hypermutated colon cancer to fewer than one per sample in low-mutation cancer types. To further confirm this result, we performed a targeted peptide search using PepQuery (25), a peptide-centric search tool, to investigate the presence of well-known mutation-derived SAAVs. We found no evidence for them, even for the most common missense mutations, such as KRAS p.G12D in pancreatic cancer (see Supplemental Note for additional information). Therefore, the PDX proteomics data are unlikely to contain somatic mutation–derived SAAVs, and any potential false positives of cross-species SAAVs should be attributed to the methods under assessment.

### Identifying Cross-Species SAAVs (“TE SAAVs”) Using Open Search Strategies

**Fig. 3:**
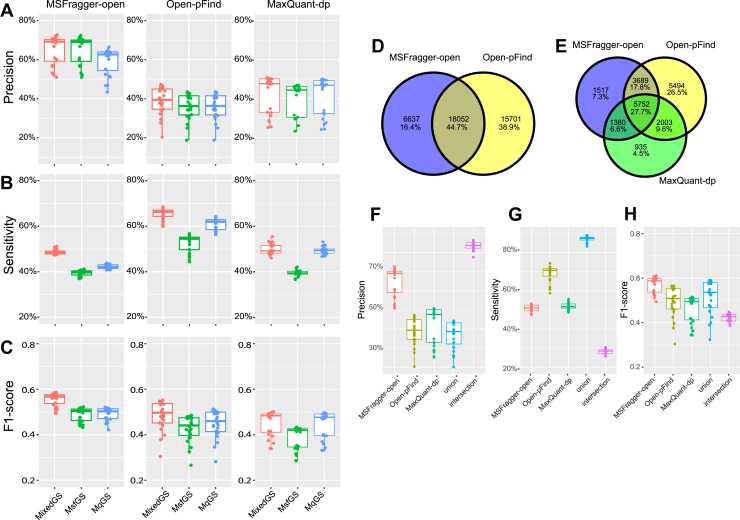
**Performance of open search strategies in identifying cross-species SAAVs.***A*–*C,* box plots showing the precision (*A*), sensitivity (*B*), and F1 score (*C*) of three open search tools at the PSM level. For each box plot, three types of gold-standard PSMs were used individually: mixed gold-standard PSMs (MixedGS), gold-standard PSMs from the MouseDb-informed search using MaxQuant (MqGS), and gold-standard PSMs from the MouseDb-informed search using MSFragger (MsfGS). *D* and *E,* Venn diagrams illustrating the overlap of correctly identified PSMs between MSFragger-open and Open-pFind (*D*) or among all three tools (*E*). *F*–*H,* box plots showing the precision (*F*), sensitivity (*G*), and F1 score (*H*) of the three tools, as well as their intersection and union. Precision and sensitivity were calculated based on mixed gold-standard PSMs. PSM, peptide-spectrum match; SAAV, single amino acid variation.

For real-world TE-related SAAV identification, MS open search strategies do not assume specific SAAVs and are theoretically the most suitable approach for studying TEs with limited prior knowledge. We first tried three recently published open search tools, MSFragger (the open search mode, hereafter referred to as “MSFragger-open”) (33), Open-pFind (34), and MaxQuant (the dependent peptide search mode, hereafter referred to as “MaxQuant-dp”), to identify the PSMs with cross-species SAAVs. While Open-pFind directly outputs inferred SAAVs, the results from MSFragger-open and MaxQuant-dp provide only the mass difference between original peptides and those with PTMs. Nonetheless, they can be translated to potential SAAVs with additional result parsing (see the *Experimental procedures* section). Based on mixed gold-standard PSMs, MSFragger-open achieved a precision of 50.9% to 72.7% (median, 69.2%), a sensitivity of 47.3% to 51.1% (median, 48.3%), and an F1 score of 0.493 to 0.586 at the PSM level. Open-pFind achieved a precision of 20.3% to 47.2% (median, 39.4%), a sensitivity of 59.9% to 68.4% (median, 66.2%), and an F1 score of 0.304 to 0.552 (median, 0.497) at the PSM level. For MaxQuant-dp, following its assumption, the ground truth was restricted to mouse peptides whose cognate human peptides were also expressed in the same PDX sample. Under this condition, MaxQuant-dp achieved a precision of 25.2% to 59.8% (median, 47.7%), a sensitivity of 46.0% to 55.4% (median, 49.1%), and a F1 score of 0.338 to 0.501 (median, 0.483) (Fig. 3, *A*–*C*). Performance comparisons across the three tools were consistent in the prostate cancer dataset (Supplemental Fig. S2, *A*–*C*), although their performance was lower, possibly because of lower data quality. These results suggest that MSFragger-open offers the highest precision and overall the best performance, whereas Open-pFind provides the highest sensitivity.

We next examined the overlap of PSMs correctly identified by these search tools. Among all the PSMs correctly identified, 44.7% were commonly detected both by MSFragger-open and Open-pFind (Fig. 3*D*). For the PSM subset corresponding to the ground truth of MaxQuant-dp, only 27.7% were identified by all the three tools (Fig. 3*E*). The union of the three tools achieved a sensitivity of 79.7% to 88.2% (median, 86.2%) sensitivity at compromised precision, whereas their intersection yielded a precision of 66.2% to 80.1% (median, 78.8%) precision at compromised sensitivity (Fig. 3, *F* and *G*). Notably, neither the union nor the intersection achieved an improved F1 score compared with MSFragger-open, indicating that they should be adopted for improving either sensitivity or precision as specific priorities, rather than as a general strategy (Fig. 3*H*). Similar observations were found from the prostate cancer dataset (Supplemental Fig. S2, *D*–*G*).

### Differential Precision Rates Across Identified SAAVs

**Fig. 4:**
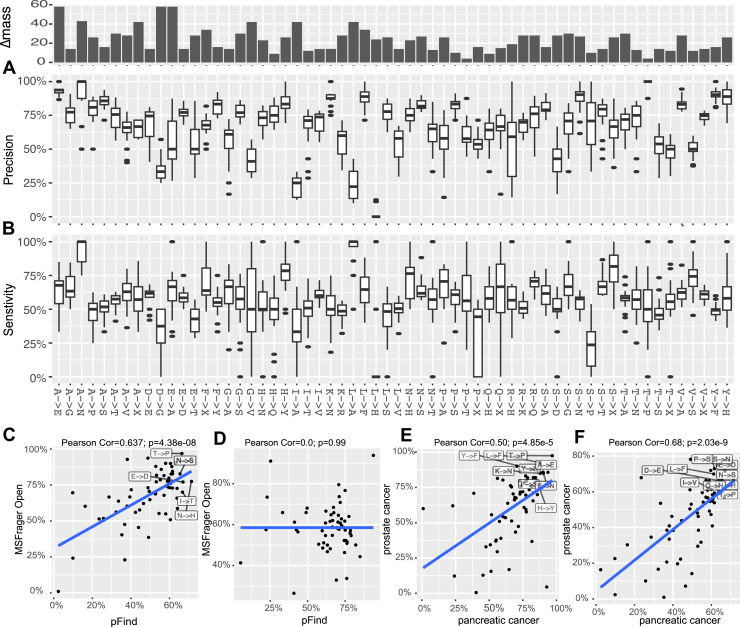
**Differential identification of SAAVs.***A* and *B,* box plots showing the precision (*A*) and sensitivity (*B*) of MSFragger-open in identifying different SAAVs. Mass shifts associated with each SAAV are displayed in the *top* bar plot. *C* and *D,* correlations of precision (*C*) and sensitivity (*D*) in identifying different SAAVs using MSFragger-open and Open-pFind. Representative SAAVs that can be accurately identified by both search methods are highlighted. *E* and *F,* correlations of precision in identifying different SAAVs between the two cancer datasets using MSFragger-open (*E*) and Open-pFind (*F*). Representative SAAVs that can be accurately identified in both datasets are highlighted. PSM, peptide-spectrum match; SAAV, single amino acid variation.

We next conducted a more detailed characterization to gain potential insights into the current performance. First, we observed that not all the SAAVs were identified with the same sensitivity or precision (Fig. 4, *A* and *B*). For instance, the identification of H(His)→S(Ser), T(Thr)→P(Pro), and Q(Gln)→N(Asn) achieved high precision for both MSFragger-Open and Open-pFind. The differential precision rates for identifying SAAVs were significantly correlated between the tools (*p* < 0.001, Fig. 4*C*). Moreover, the precision rates across these SAAVs are also consistent between the two cancer datasets (*p* < 0.001, Fig. 4, *E* and *F*). These findings suggest that the downstream analyses need to be mindful of differential precisions among the SAAVs. Interestingly, the sensitivity for the same SAAV varied between different tools, indicating potential for enhancing discovery by leveraging multiple tools (Fig. 4*D*).

We next investigated if the differential performance in identifying different SAAVs is related to the mass difference caused by the AA substitution. Our analysis revealed no significant correlation between the precision or sensitivity and mass difference for the SAAVs (Supplemental Fig. S3, *A*–*D*). This finding is not unexpected, as the resolution of advanced MS already exceeds the mass difference associated with most SAAVs. The key factors determining the differential precision across SAAVs remain unclear. Possible explanations may include chemical properties, the presence of isotopes, or the influence of sequence context.

### Further Approaches to Refine Open Search Results

**Fig. 5:**
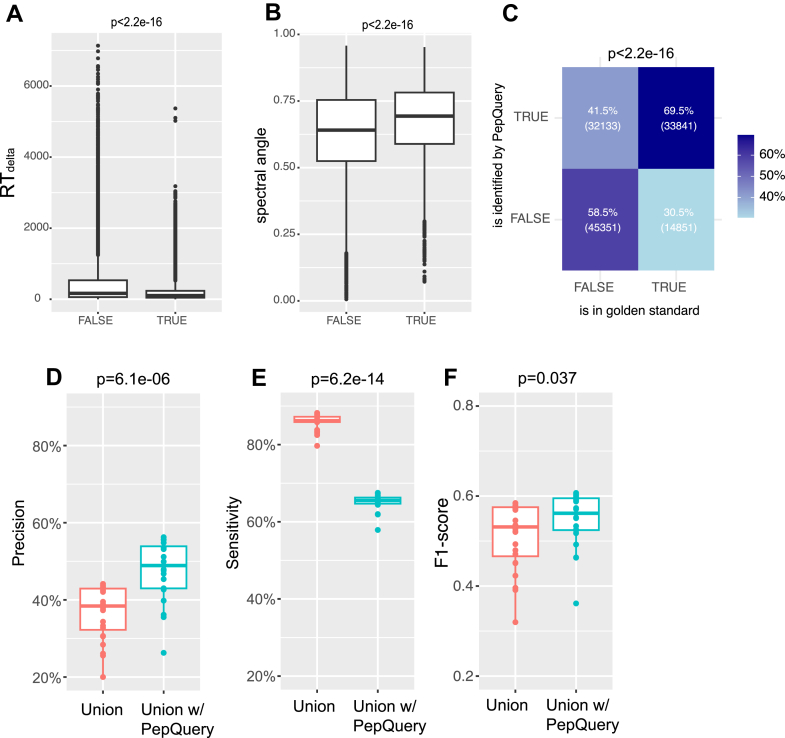
**Assessing PSM quality using third-party tools.***A,* box plot showing the absolute difference between experimental retention times (RTs) and predicted RTs for peptides from correctly identified (TRUE) or incorrectly identified (FALSE) PSMs, based on mixed gold-standard PSMs. *B,* box plot showing the spectral angles between experimental and predicted spectra for peptides from the two PSM groups in (*A*). *C,* a two-by-two table illustrating the overlap between PepQuery-identified and gold-standard peptides. *D*–*F,* alteration of precision (*D*), sensitivity (*E*), and F1 score (*F*) when applying PepQuery to the union of open search results, based on mixed gold-standard PSMs. PSM, peptide-spectrum match.

Next, we asked whether the true or false identifications could potentially be probed by third-party tools employing algorithms orthogonal to the open search. First, we assessed the quality of PSMs by autoRT (35) and pDeep2 (36), which leverage deep learning (DL) models to predict RT and spectra, respectively, based on the search engine–resolved peptide sequences. The similarity between the sequence-derived RTs or spectra and their experimental counterparts has been used as a metric for evaluating PSM quality and search engine performance (37, 38). We found that PSMs with correctly identified SAAVs significantly outperformed those with incorrectly identified SAAVs on these DL-derived evaluation metrics (*p* < 0.001, Wilcox’s rank test, Fig. 5, *A* and *B*). This finding suggests that the quality of PSMs inferred by DL models could potentially serve as a mechanism to further enhance the precision of SAAV identification.

Furthermore, we evaluated the PSMs using PepQuery, a peptide-centric search tool, designed to verify the presence of specific peptides (25). PepQuery employs an extensive competitive filtering mechanism to assess the likelihood of candidate peptides, making it suitable for evaluating the quality of identified SAAVs. For the open search results, the PepQuery confirmation rate for the peptides with correctly identified SAAVs was substantially higher than for those with incorrectly identified SAAVs (*p* < 0.001, one-tailed Fisher’s exact test, Fig. 5*C*). Applying PepQuery confirmation on top of the union of open search results increased the precision of identification (median rate, 38.4% *versus* 48.9%, *p* < 0.001) (Fig. 5*D*). However, this came at the cost of significantly reduced sensitivity (median rate, 85.2% *versus* 65.5%), albeit slightly improved F1 score (median, 0.533 *versus* 0.566, *p* = 0.037) (Fig. 5, *E* and *F*). Thus, while PepQuery is more likely to confirm correct PSMs with SAAVs than incorrect ones, it is not yet suitable for integration with open search tools to improve SAAV identification strategies. Nevertheless, all these third-party tools can refine open search results if downstream analyses prefer higher confidence over the comprehensiveness of identified SAAVs.

### Closed Search With Predefined SAAVs

Finally, we evaluated the likelihood of identifying SAAVs using a conventional closed search, which assumes that PTMs are specific and known. It is worth noting that, without strong prior knowledge of the SAAV types present in the biological sample, such closed search strategies are impractical to implement. This is because the number of possible SAAVs (*i.e.*, the 20 × 20 combinations of AA substitutions) far exceeds the search space capabilities of these methods.

**Fig. 6:**
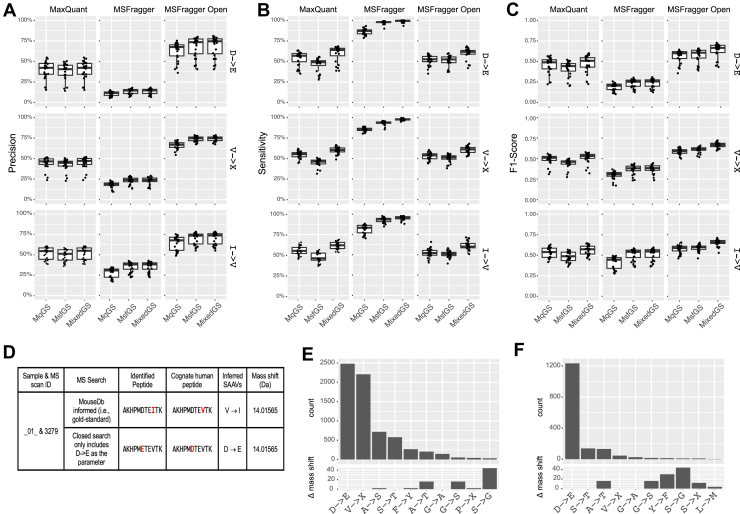
**Performance of closed search with preset PTMs in identifying cross-species SAAVs.***A*–*C,* box plots showing the precision (*A*), sensitivity (*B*), and F1 score (*C*) of closed searches by MaxQuant and MSFragger in identifying three SAAVs: D→E (*top*), V→X (*middle*), and I→V (*bottom*). For each plot, the three types of gold-standard PSMs are used: gold-standard PSMs from the MouseDb-informed search using MaxQuant (MqGS) or MSFragger (MsfGS) or the mixed gold-standard PSMs (MixedGS). The MSFragger open-search results are included for reference. *D,* example of incorrect SAAV identification in a closed search specifying only D→E. In this example, the gold-standard mouse peptide is AKHPMDTEITK, with the SAAV V→I; however, as V→I is not included in the search parameters (*i.e.*, lack of competition), the closed search incorrectly assigns D→E to this PSM at a different site. *E* and *F,* bar plots showing the counts of true SAAVs identified or misidentified by the closed search as D→E. The differences in mass shift between D→E and each of the misidentified SAAVs (Δ mass shift) are indicated at the *bottom*. The closed search was performed using MSFragger (*E*) or MaxQuant (*F*). PSM, peptide-spectrum match; PTM, post-translational modification; SAAV, single amino acid variation.

We selected the three most abundant SAAVs identified from the open search (*i.e.*, D(Asp)→E(Glu), V(Val)→X(Leu or Ile), and I(Ile)→V(Val)) to assess how effectively the closed search could identify them. To this end, we performed closed searches using MSFragger and MaxQuant against the human protein database, setting each SAAV as a variable PTM parameter. Unexpectedly, the closed search significantly underperformed the open search in precision across all three SAAV types (Fig. 6*A*). For instance, although MSFragger exhibited the highest precision among the three tools in the open search, its closed search yielded median precision values of only 14.7%, 24.0%, and 38.2% for the three SAAVs, respectively, compared with 74.5%, 74.8%, and 73.8% from the open search (*p* < 0.001, Wilcoxon rank-sum test). The MaxQuant closed search also underperformed the open search in precision, though to a lesser extent (Fig. 6*A*). Similar results were found from analyzing the prostate cancer dataset (Supplemental Fig. S4*A*). As the sensitivity was less likely to be a concern (nearly 100% for MSFragger, Fig. 6*B* and Supplemental Fig. S4*B*), the primary limitation of closed search strategies stemmed from the high rate of false-positive SAAVs (Fig. 6*C* and Supplemental Fig. S4*C*).

We next examined the PSMs contributing to false-positive SAAVs and found that most of them resulted from PTM site mislocalization by the search tools. Specifically, the search tools successfully matched the spectra to correct base peptides but assigned the PTM (*i.e.*, mass shift) to an incorrect position. The true SAAVs, which were not specified in the search parameters, were located at a different site on the peptide (Fig. 6*D*). As expected, the SAAVs in these incorrect PSMs from the closed search and those in the gold-standard PSMs exhibited identical or very similar AA mass shift (Fig. 6, *E* and *F*, Supplemental Fig. S4, *D* and *E*). These results suggest that, in the absence of peptides with correct SAAVs in the reference database to compete for PSMs, the search tools tend to overconfidently assign the preset PTMs, thereby compromising precision. Consequently, the closed search may not be suitable when multiple AA substitutions occur, a scenario not uncommon in complex diseases where protein translation can be systematically altered. It is also noteworthy that mitigating PTM mislocalization, which has been investigated in other types of protein modifications, such as phosphorylation (38), would be a key factor to improve the precision of TE-SAAV identification in closed searches.

## Discussion

The protein translation process is highly regulated, and its disruption is implicated in many diseases (7). Despite improvements in both MS proteomics data generation and processing, it remains unclear how effectively SAAVs resulting from TEs can be identified in current MS data. Our research leverages AA substitutions between mouse and human, identified in PDX MS data, for this evaluation.

Compared with previous studies (25, 27) that relied on AA alteration resulting from somatic or germline mutations, our ground truth setting offers several key advantages. First, it better reflects the patterns of SAAVs caused by TEs. Moreover, since substitutions between biochemically similar AAs are less likely to cause protein degradation (39, 40, 41), our TE simulation using PDX samples captures TE SAAVs that are more likely to be retained in the proteome. Second, SAAVs in the PDX data are identified directly from proteomics, whereas genomic mutations are not guaranteed to be expressed at the protein level. Finally, SAAVs from PDX data enable PSM-level evaluation, providing valuable feedback to tool developers for optimizing their tools. For instance, our analysis of closed search results identifies PTM localization as a key bottleneck in identifying TE-SAAVs using this search strategy.

Conversely, our ground truth is limited by potential incorrect PSM identifications derived from MouseDb-informed search, as evidenced by tool inconsistency—a result expected given that no search tools are perfect. However, as a flawless gold-standard PSM set can only be obtained from synthetic peptides, which cannot match the diversity of PDX datasets, our ground truth still represents a valuable resource for this benchmarking.

Our results show that while performance varies by SAAV type, current open-search tools can achieve an overall sensitivity above 65% and precision above 70% for SAAVs in high-quality MS data. Open-pFind outperforms other tools in sensitivity, whereas MSFragger yields the best precision. Notably, even for predefined SAAVs, which can only be hypothesized with prior knowledge, open-search tools performed better than fixed-search methods. We found that closed search methods with preset SAAVs produce a large number of false-positive identifications, primarily because of PTM mislocalization when the ground truth PTMs are not included in the search database.

The results from different open-search tools partially overlapped, providing a degree of complementarity. For instance, combining results can increase sensitivity, whereas focusing on their intersection can enhance precision. Depending on the stringency required for downstream mechanistic studies, one might use post-processing tools, such as PepQuery, to further filter open-search results and obtain a high-confidence SAAV set.

Interestingly, the variation in precision across SAAVs appears independent of the tools or datasets used, suggesting that other factors, such as biochemical properties or sequence context, may influence their identification in MS data. We propose that dedicated machine learning or DL studies could provide further insights into this issue. In particular, protein sequence-based DL models offer promising opportunities for processing and analyzing proteomics data (42), aligning with the critical need to identify TE-SAAVs that exist solely at the protein level.

In summary, by using the abundant high-confidence SAAVs from PDX MS data as a ground truth, our evaluation of TE identification at the PSM level provides insights into the performance of current MS search tools, the reliability of downstream functional exploration of TEs, and potential strategies for tool optimization.

## Data Availability

All the proteomics data, genomic data, and gene annotations are publicly available, as indicated above. Data analyses were performed by R (version 4.4.0). The codes used for the study are available at https://github.com/HuangLabAtUAB/translationalError. The PSM-level SAAVs from MouseDb-informed searches, open searches, and closed searches are available at https://zenodo.org/records/17101732, with annotations in the Supplemental Note.

## Supplemental Data

This article contains supplemental data (18, 25, 35, 43, 44, 45, 46, 47).

## Conflict of Interest

The authors declare no competing interests.

## Supplementary data

Supplemental Figure

Supplemental Note
