# Supplementary material for: Assessing the Performance of Mass Spectrometry Search Strategies in Identifying Translational Errors Using PDX Proteomics Data
Source: Mol Cell Proteomics. 2025 Dec 22;25(2):101500. doi: 10.1016/j.mcpro.2025.101500 (PMC12856148; doi:10.1016/j.mcpro.2025.101500)
Supplement: Supplemental Figure [file mmc1.docx]

**
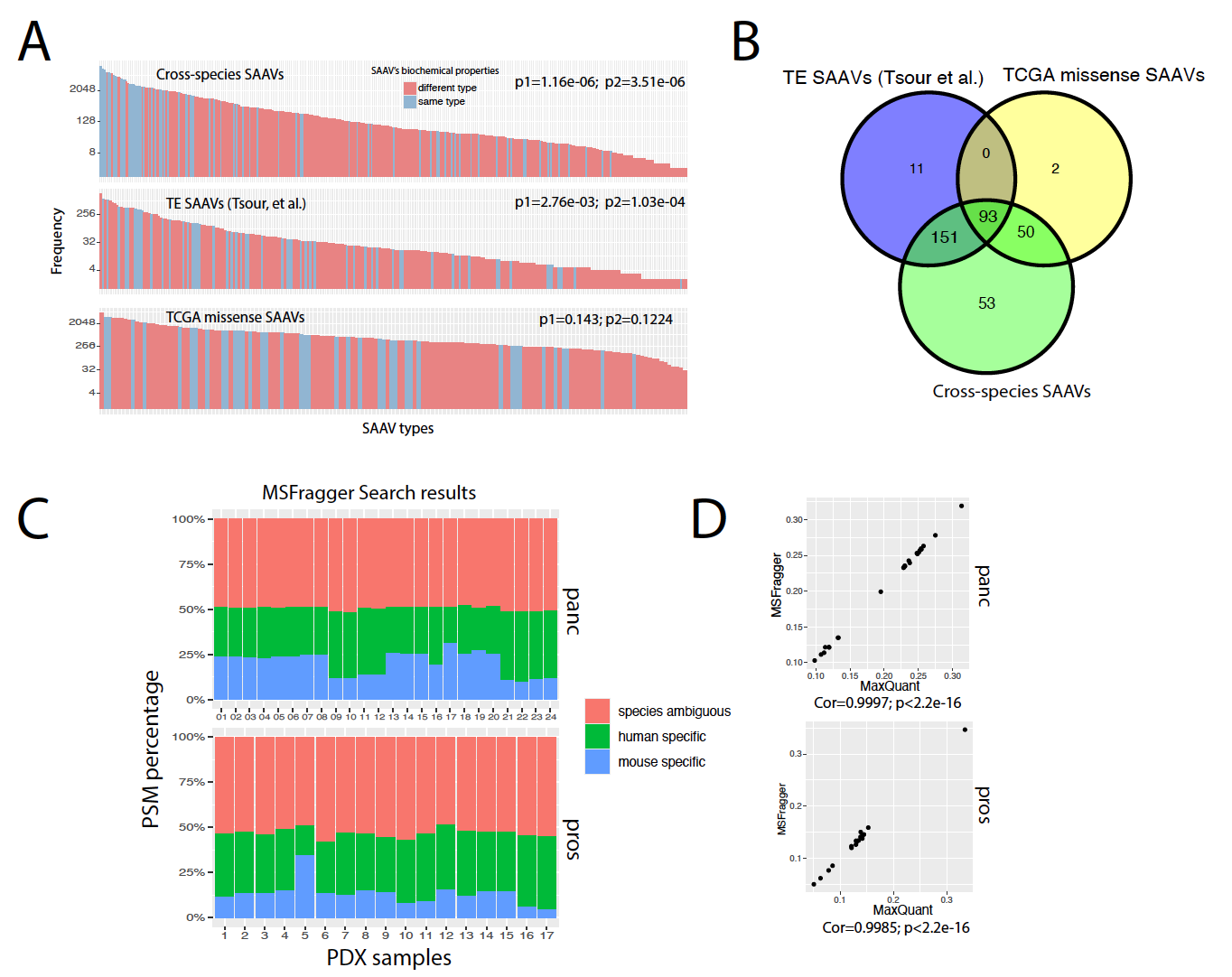
**

**Supplemental Figure 1. The MouseDb-informed search.** (**A**) Comparison of the SAAV landscape for human-mouse cross-species SAAVs, TE-SAAVs, and TCGA missense SAAVs. Bar plots display the counts of each SAAV type, ranked from most abundant to least abundant. The p-values were derived from the Wilcoxon test (p1) or Kolmogorov-Smirnov test (p2) to assess whether SAAVs of each type are enriched with similar biochemical properties. (B) Venn diagram showing the overlap of unique SAAVs from the three cohorts. Among the 255 TE SAAV types, 244 of them (95.7%) were present in the cross-species SAAVs, whereas only 93 of them (36.4%) overlapped with the TCGA missense SAAVs. (C) Sample-wise composition of human and mouse PSMs from the mouseDb-informed MSFragger search for the two datasets. Colors denote PSMs with different species specificity. (**D**) Scatter plots showing correlations between the two tools in identifying the percentage of mouse-specific PSMs in the pancreatic cancer (panc, upper) and the prostate cancer (pros, lower).

**
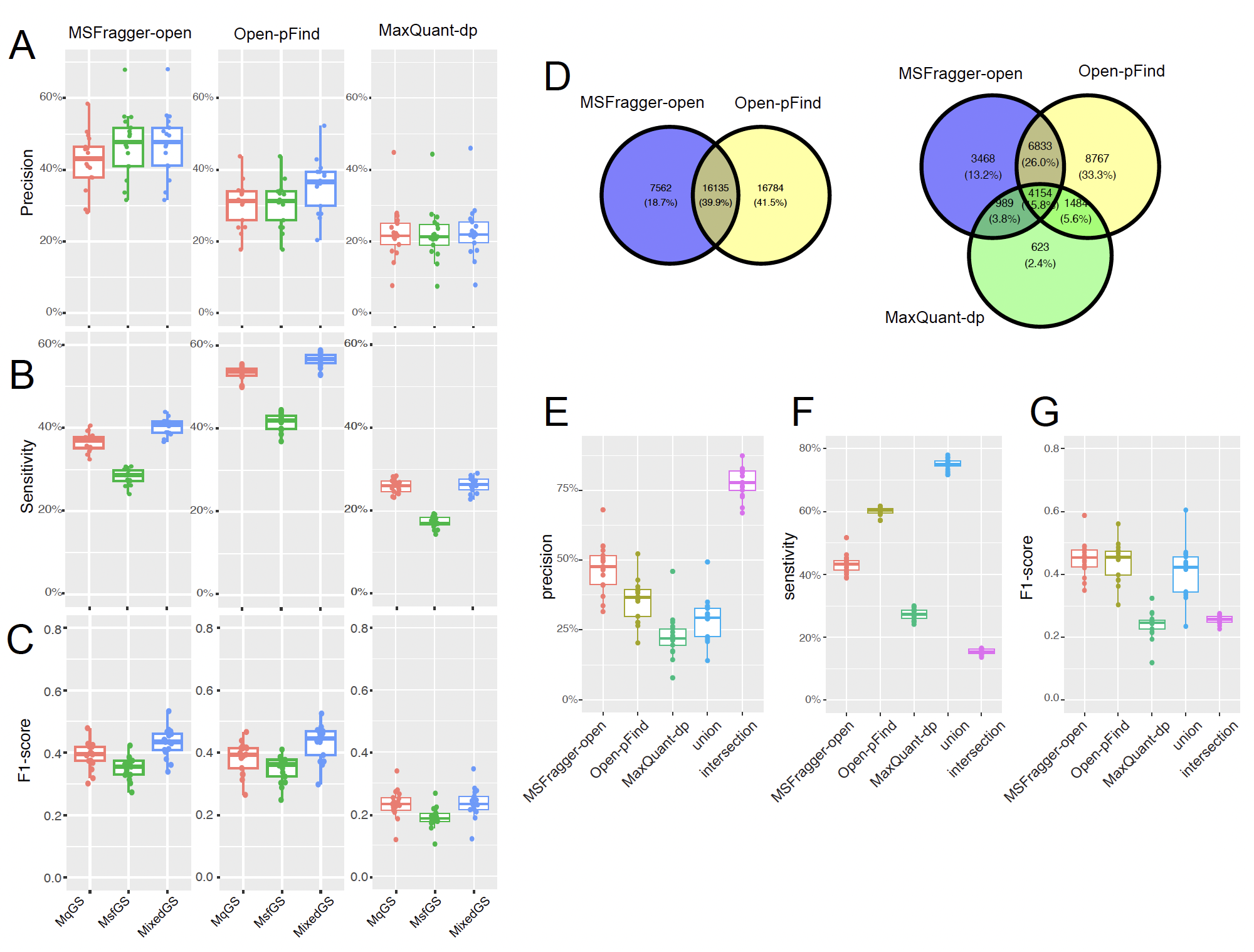
Supplemental Figure 2. Performance of open search strategies in identifying cross-species SAAVs for the prostate cancer datasets.** (**A–C**) Box plots showing the precision (A), sensitivity (B), and F1 score (C) of three open search tools at the PSM level. For each box plot, three types of gold-standard PSMs were used individually: mixed gold-standard PSMs (MixedGS), gold-standard PSMs from a mouseDb-informed search using MSFragger (MsfGS), and gold-standard PSMs from MaxQuant (MqGS). (**D**) Venn diagrams illustrating the overlap of correctly identified PSMs between MSFragger-open and Open-pFind (left) or among all three tools (right). (**E–G**) Box plots showing the precision (E), sensitivity (F) and F1 score (G) of the three tools, as well as their intersection and union. Precision and sensitivity were calculated based on mixed gold-standard PSMs.


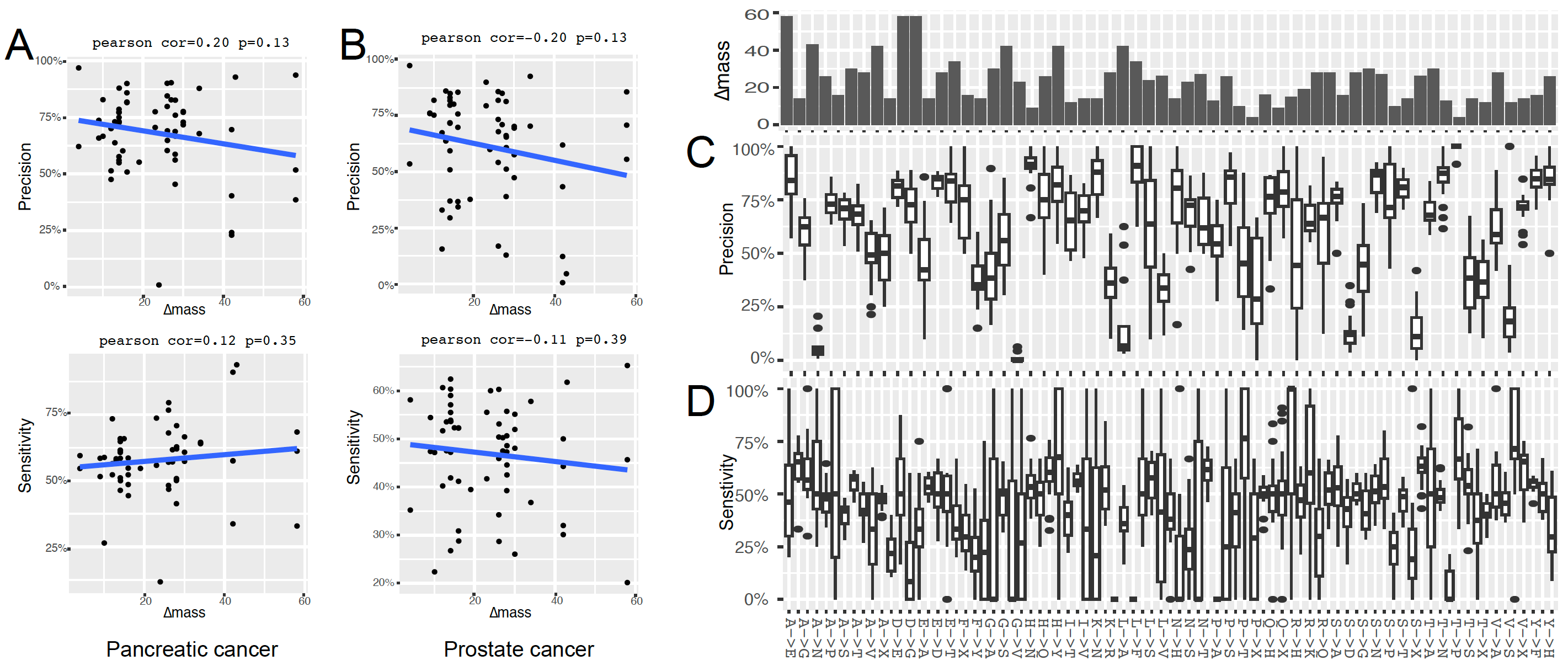


**Supplemental Figure 3. The difficulty in identifying individual SAAVs is independent of mass shift.** **(A–B)** Correlation between the mass shift of each SAAV and its identification precision (upper panel) or sensitivity (lower panel). Panel A represents the pancreatic cancer dataset, and Panel B represents the prostate cancer dataset. **(C–D)** Box plots showing the precision (C) and sensitivity (D) of MSFragger-open in identifying different SAAVs in the prostate cancer dataset. The mass shifts associated with each SAAV are displayed in the top bar plot.


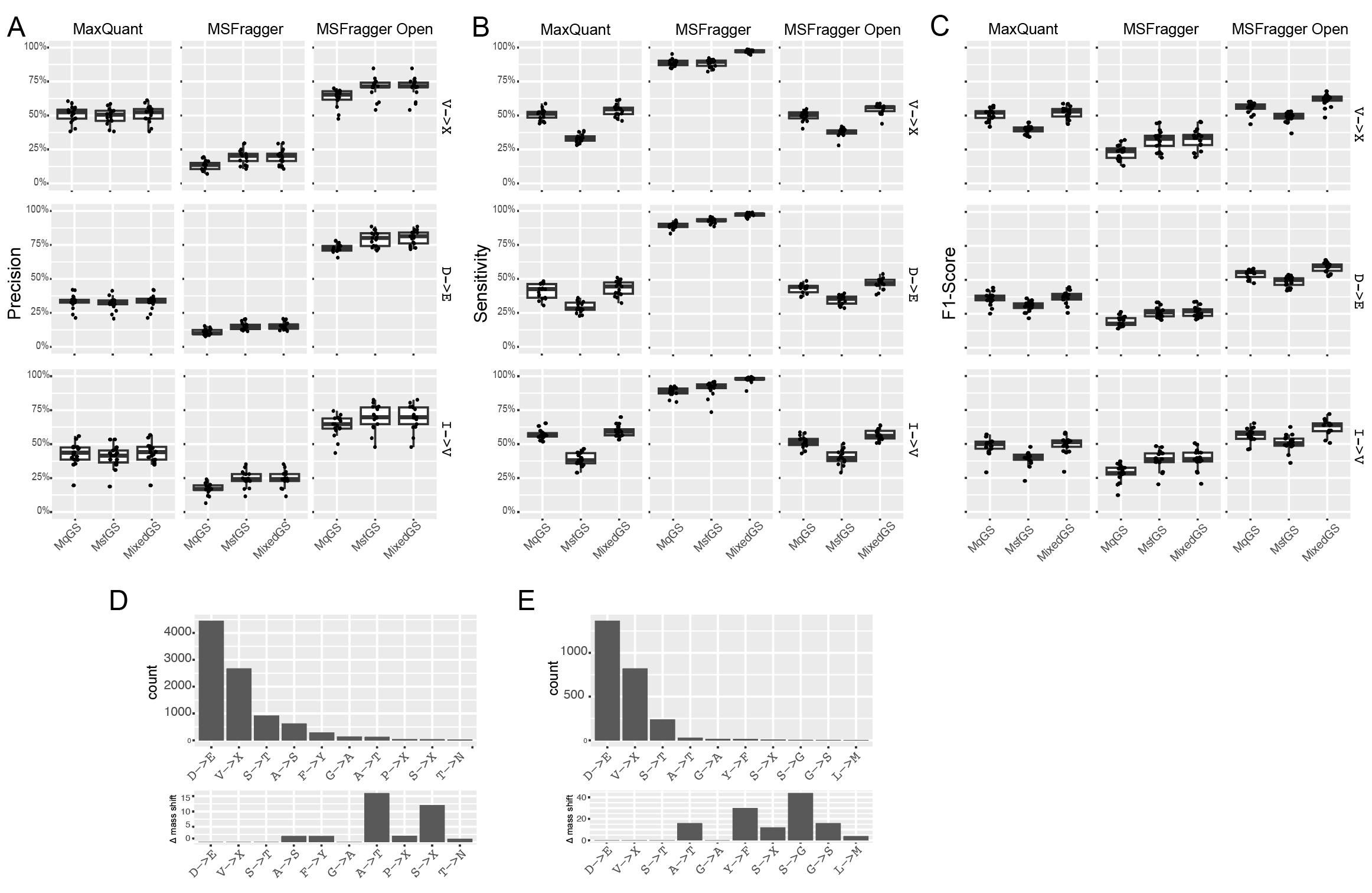


**Supplemental Figure 4. Performance of closed search with preset PTMs in identifying cross-species SAAVs for the prostate cancer dataset.** (**A–C**) Box plots showing the precision (A), sensitivity (B) and F1 score (C) of closed searches by MaxQuant and MSFragger in identifying three SAAVs: V→X (top), D→E (middle), and I→V (bottom). For each plot, the three types of gold-standard PSMs were used: gold-standard PSMs from the mouseDb-informed search using MaxQuant (MqGS) or MSFragger (MsfGS), or the mixed gold-standard PSMs (MixedGS). (**D–E**) Bar plots showing the counts of frequent SAAVs misidentified by the closed search when identifying D→E. The differences in mass shift between D→E and each of the misidentified SAAVs (Δ mass shift) are indicated at the bottom. The closed search was performed using MSFragger (D) or MaxQuant (E).
