# Supplementary material for: Assessing the Performance of Mass Spectrometry Search Strategies in Identifying Translational Errors Using PDX Proteomics Data
Source: Mol Cell Proteomics. 2025 Dec 22;25(2):101500. doi: 10.1016/j.mcpro.2025.101500 (PMC12856148; doi:10.1016/j.mcpro.2025.101500)
Supplement: Supplemental Note [file mmc2.docx]

**Supplemental Note**

**Part I: A deeper examination of the discrepancy of MouseDb-informed research from MaxQuant and MSFragger**

To investigate whether the moderate overlap between MSFragger and MaxQuant resulted from increased proteome complexity due to combining two species, we compared the tools’ search results using proteomics data from mouse-only (a mouse breast cancer cell line, **PXD036701**) and human-only (NCI60 cancer cells, **PXD005940**) datasets (**Fig SN1**). The results showed median overlap rates of 46.7% and 67.3% for the mouse and human datasets, respectively, suggesting that the differences between the two tools are not due to PDX samples but rather to their distinct algorithms for scoring peptide-spectrum matches (PSMs) and post-processing (e.g., MSFragger’s use of Percolator). Consistent with our findings in **Fig 2D**, MSFragger identified most non-overlapping PSMs.


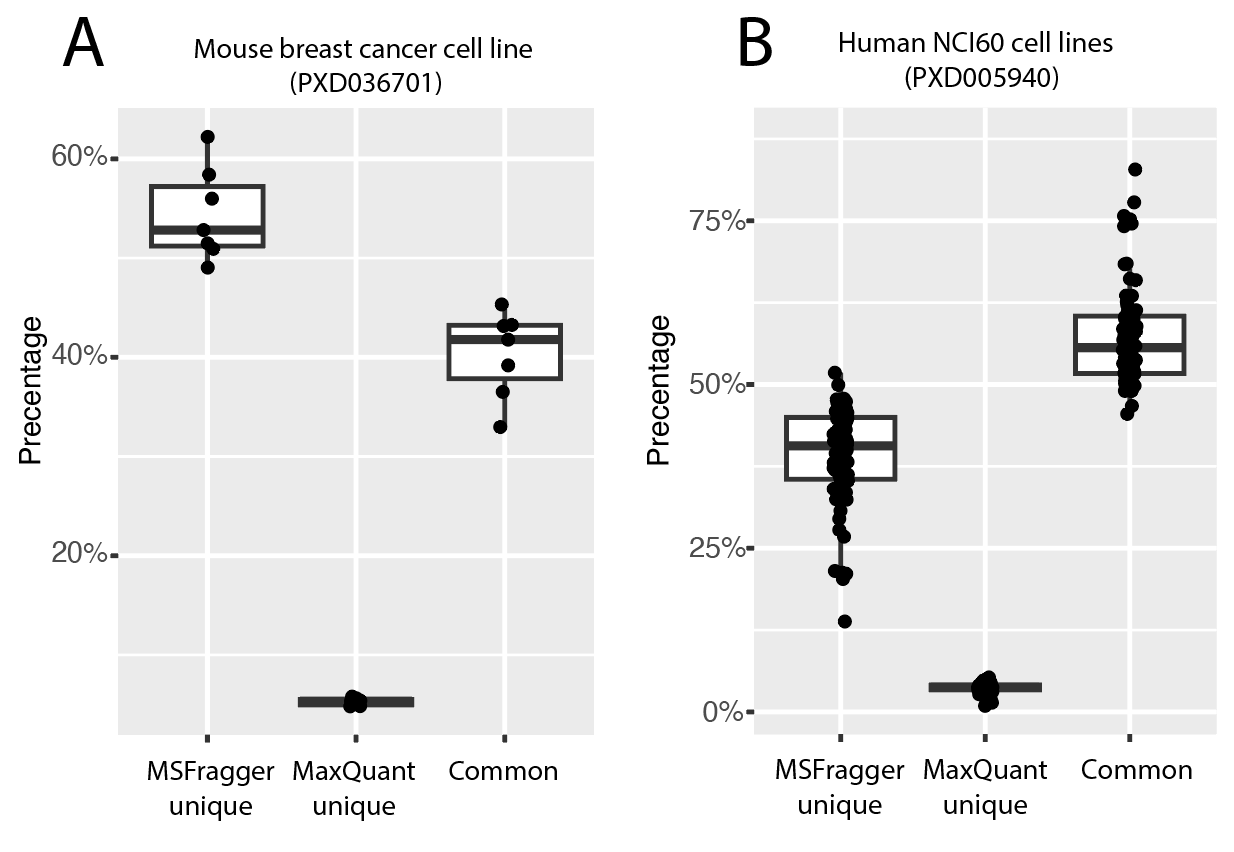


**Figure SN1**. Percentages of PSMs in tool-unique or tool-overlapped groups. Results were generated from mouse (A) or human (B) proteomics data.

Partial overlap between search tools has been reported previously. For instance, a recent study comparing different search tools reported that MSFragger and MaxQuant accounted for 72% and 50% of the total union of unique peptides, respectively (1). These findings align with our results, indicating moderate overlap and greater identification by MSFragger than MaxQuant. Similarly, another study involving six tools (Open-pFind, Comet, MS-GF+, X!Tandem, SysteMHC, and MaxQuant) for immunopeptidome analysis found that only 30–50% of search results from a single tool overlapped with those from other tools (2).

As expected, PSMs overlapping between the two tools had significantly higher quality scores than tool-unique PSMs (**Fig SN2**). Therefore, in our mixed gold standard, we used overlapping PSMs to evaluate the quality of open- and closed-search strategies, minimizing the risk of incorrectly dismissing PSM candidates from these strategies.


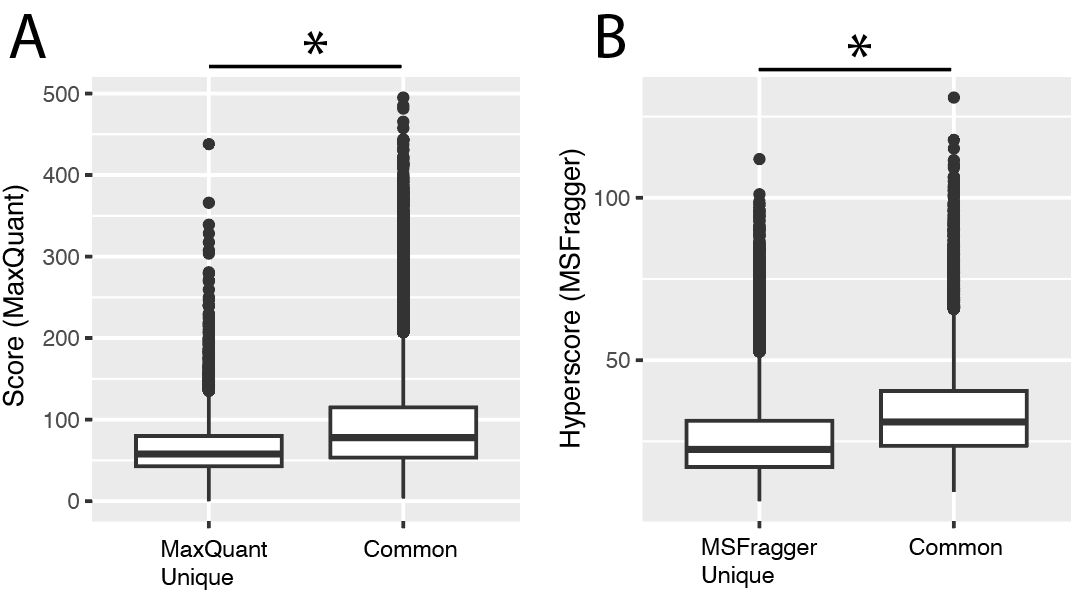


**Figure SN2**. Scores of PSMs identified by MaxQuant (A) and MSFragger (B). For each tool, the PSMs are grouped as tool-unique or tool-overlapped (* p<0.001).

Furthermore, to gain additional insight into the reliability of the search results, we analyzed matched RNA-seq data from prostate cancer samples (<https://www.ebi.ac.uk/ena/browser/view/PRJEB9660>) (3). Although protein and RNA expression are not always well-correlated for specific genes, as shown in recent proteogenomic studies, most genes with expressed proteins are still expected to have detectable RNA. We found that genes with proteins detected by both tools had significantly higher RNA expression than those detected by only one tool or not detected at all (**Fig SN3**). Interestingly, although MSFragger identified more PSMs and proteins, the RNAs corresponding to MSFragger-unique proteins showed slightly higher expression than those for MaxQuant-unique proteins (median log2 FPKM 4.30 vs. 4.08, not significant), suggesting that RNA expression supports the additional genes identified by MSFragger. Thus, while RNA expression corroborates that overlapping proteins are of higher quality, it does not provide sufficient evidence to exclude tool-unique PSMs from the gold standard.


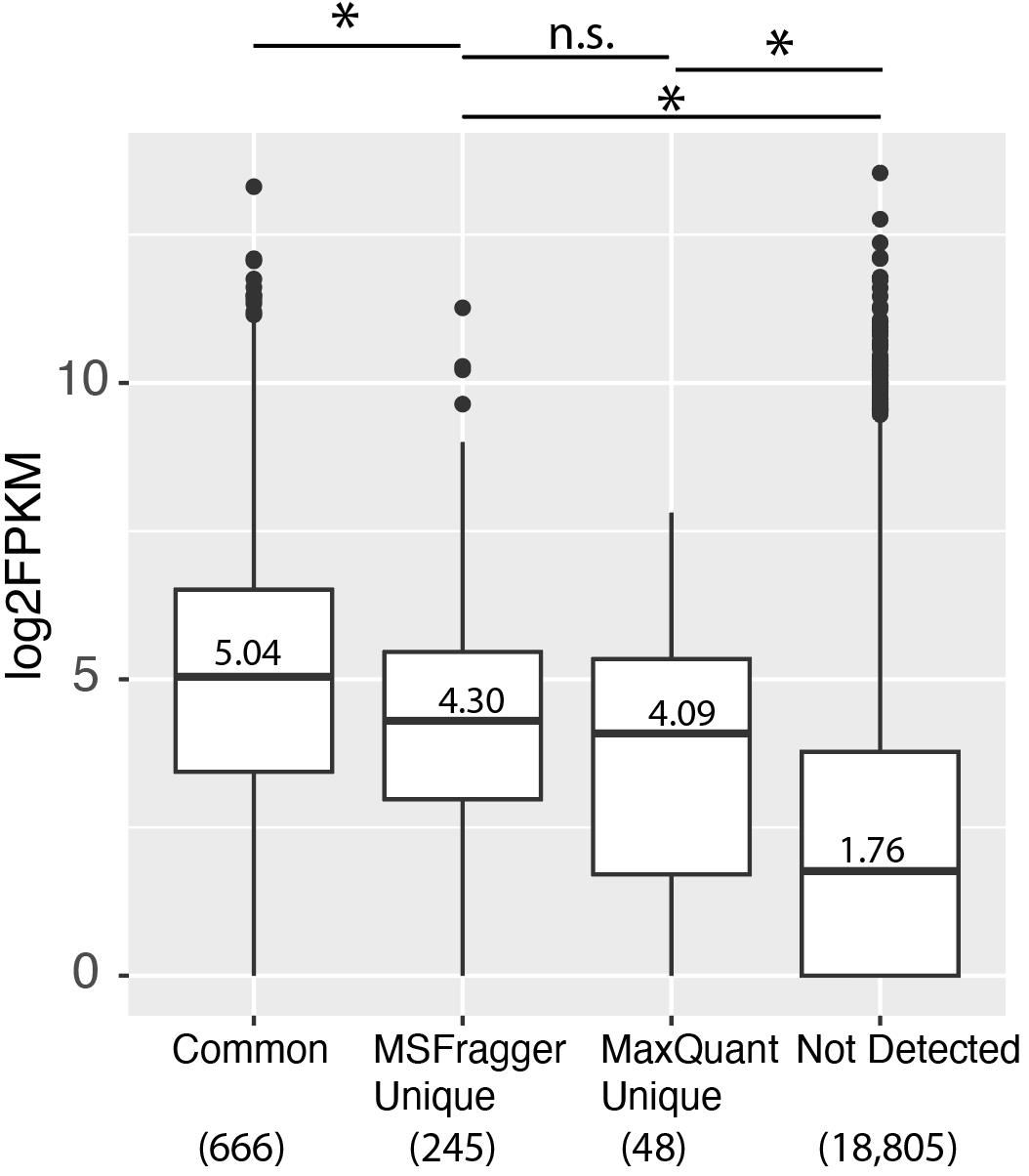


**Figure SN3**. RNA expression (log2FPKM) across gene groups with different protein identification evidence. Numbers in parentheses indicate the number of genes in each group (*p < 0.01, n.s. not significant).

In summary, the modest overlap between the two tools is not caused by the concatenated human and mouse proteome but is likely due to differences in the tools’ inherent algorithms, as supported by other studies (1, 2). Quality scores and matched RNA-seq data indicate that overlapping PSMs are of higher quality than tool-unique PSMs. However, neither metric provides sufficient evidence to exclude any tool-unique PSMs from the gold standard. Therefore, we defined the ‘mixed’ gold standard using overlapping PSMs to minimize the risk of incorrectly dismissing PSM candidates.

**Part II: A deep analysis of SAAV sources of the PDX proteomics data.**

Since we used cancerous samples to benchmark SAAVs, there is a possibility that the mutation-related SAAVs would confound our interpretation of cross-species SAAVs. Below, we provide evidence to examine the existence of mutation SAAVs.

1. For both cancer types (PDAC and prostate cancer), none of the somatic mutations reported by TCGA and CPTAC are overlap with the cross-species SAAVs.

2. To identify potential SAAVs caused by mutations, we performed MS searches using a customized database strategy where the TCGA- and CPTAC-reported high quality mutation-resulted SAAV candidates were added into the search database (4,253 for PDAC and 8,318 or prostate cancer). Moreover, for the prostate cancer PDX samples, we perform mutation calling based on the matched RNA-seq data (16,096 mutations). The mutation calling was performed using the nf-core rnadnavar pipeline (<https://nf-co.re/rnadnavar/dev/>). The customized databases were built by CustomProDBJ (4). We used MaxQuant and MSFragger for customized database search and did not identify any peptides from the proteomics data that contain mutation-related SAAVs.

We reason that lacking detection of mutation SAAVs was due to their low abundance, which can be estimated from the known proteogenomics studies. For instance, in the CPTAC colorectal cancer, the average number of peptides with mutation-related SAAVs is only ~10 per sample (5). Notably, such an identification is based on the fact that one third of colorectal cancers are microsatellite-instable (i.e., hypermutated), and that the CPTAC sample fractions are much more than the PDX proteomics data we used (fraction comparison: 15 in colorectal cancer study vs.1 in PDAC PDX). In another CPTAC study on renal clear cell cancer, a less mutated cancer type, only 32 mutation SAAVs detected from 103 cancer samples (0.31 per sample), albeit each sample being profiled with 24 HPLC fractions (6). In the CPTAC PDAC study, no peptides with mutation SAAVs were reported (7).

3. For SAAVs resulted from mutations with the highest variant allele frequency, such as KRAS G12D/R/V in PDAC and TP53 R273C/H in prostate cancer (**Table SN1**), we performed peptide-centric searches using pepQuery (8), which has demonstrated superior performance in identifying mutated peptides (4). Consistent with the results above, none were identified in our PDX proteomics data.


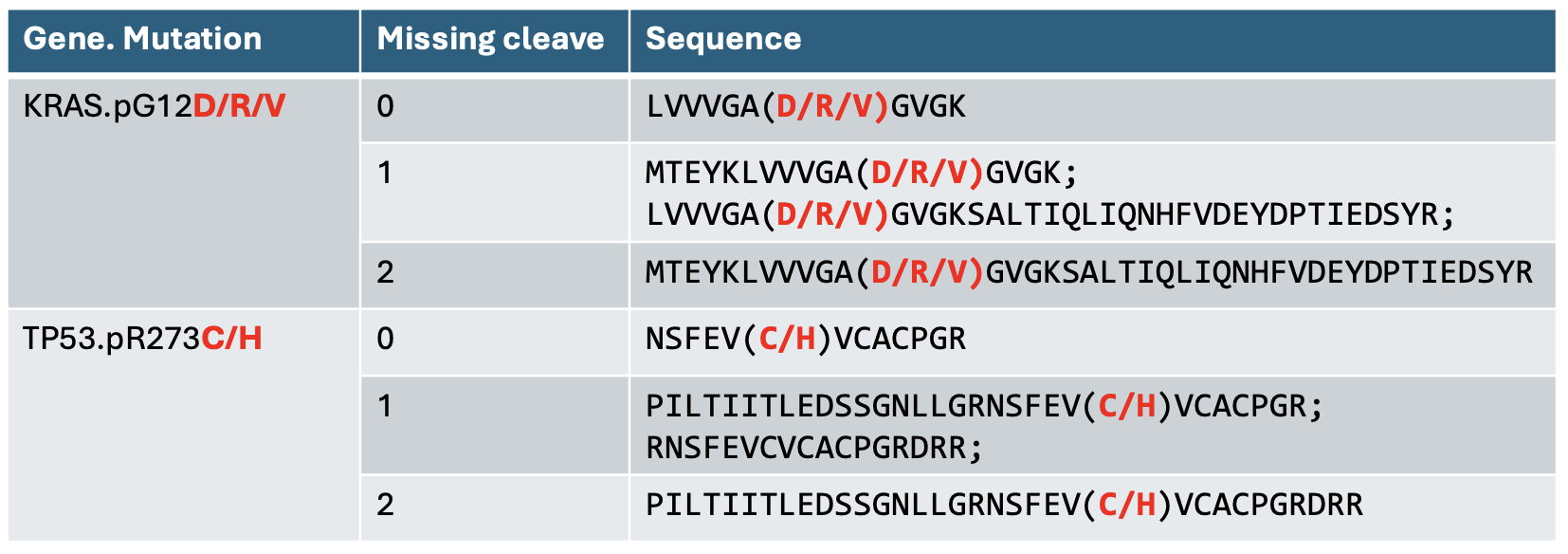


**Table SN1.** Peptides derived from the most frequent missense mutations in these two cancer types.

4. We examined our open search results, and for the SAAVs not covered in cross-species SAAVs (i.e., false positives), none of them were mutation SAAVs reported in TCGA and CPTAC large-scale cohort studies.

Taken together, although mutation-resulted SAAVs can be expressed, their low abundance and limited proteomics sensitivity make it unlikely that PDX proteomics raw data contain these signals. In other words, the PDX proteomics data closely serve as ‘normal human and mouse mix’ for this evaluation purpose.

**Part III: Supplementary files**

1. PSM-level gold-standard SAAVs identified from the mouseDb-informed search and open-search results from three tools (MSFragger-open, Open-pFind, and MaxQuant-dp) were uploaded to the Zenodo data portal at <https://zenodo.org/records/17101732>

**Table Annotations**

- The *_pdac.tsv and *_prostate.tsv files contain results from the pancreatic and prostate cancer datasets, respectively.
- MouseDbInformedSearch_msFragger.tsv and MouseDbInformedSearch_maxquant.tsv contain results from searches against the concatenated human and mouse proteome using MSFragger/FragPipe and MaxQuant, respectively. Only PSMs corresponding to mouse peptides with human-mouse SAAVs (single amino acid variants) are included. The column HumanPeptideDetected indicates whether the human cognate peptide was detected in the same sample.
- Open-pFind-results.tsv, MSFraggerOpenSearchResults.tsv, and MaxQuantDpSearchResults.tsv contain results from searches against the human proteome using the open-search tools Open-pFind, MSFragger/FragPipe (open-search mode), and MaxQuant (dependent peptide search mode), respectively.
- presetSAAV_msFragger.tsv and presetSAAV_maxQuant.tsv contain results from searches against the human proteome with SAAVs (D→E, V→X, or I→V) set as variable modifications using MSFragger or MaxQuant, respectively.

1. Protein databases used for the MS search were uploaded to the Zenodo data portal at <https://zenodo.org/records/17179272>

**Database Annotations**

- pdxHumanMouse.fasta: Concatenated human and mouse protein database.
- PDAC_mutation.tsv: Missense and indel somatic mutations for pancreatic cancer (PDAC) reported by The Cancer Genome Atlas (TCGA) and Clinical Proteomic Tumor Analysis Consortium (CPTAC).
- prostate_mutation.tsv: Missense and indel somatic mutations for prostate cancer (PRAD) reported by TCGA.
- pdxHumanPDAC.fasta: Customized human protein database incorporating PDAC somatic mutations.
- pdxHumanPROS.fasta: Customized human protein database incorporating prostate cancer somatic mutations.

**Reference**

1. Burq, M., Stepec, D., Restrepo, J., Zbontar, J., Urazbakhtin, S., Crampton, B., Tiwary, S., Chinoy, R., Miao, M., Cox, J., and Cimermancic, P. (2025) Back to Basics: Spectrum and Peptide Sequence are Sufficient for Top-tier Mass Spectrometry Proteomics Identification. *bioRxiv*, 2024.2008.2019.606805

2. Yi, X., Liao, Y., Wen, B., Li, K., Dou, Y., Savage, S. R., and Zhang, B. (2021) caAtlas: An immunopeptidome atlas of human cancer. *iScience* 24, 103107

3. Akamatsu, S., Wyatt, A. W., Lin, D., Lysakowski, S., Zhang, F., Kim, S., Tse, C., Wang, K., Mo, F., Haegert, A., Brahmbhatt, S., Bell, R., Adomat, H., Kawai, Y., Xue, H., Dong, X., Fazli, L., Tsai, H., Lotan, T. L., Kossai, M., Mosquera, J. M., Rubin, M. A., Beltran, H., Zoubeidi, A., Wang, Y., Gleave, M. E., and Collins, C. C. (2015) The Placental Gene PEG10 Promotes Progression of Neuroendocrine Prostate Cancer. *Cell Rep* 12, 922-936

4. Wen, B., Li, K., Zhang, Y., and Zhang, B. (2020) Cancer neoantigen prioritization through sensitive and reliable proteogenomics analysis. *Nat Commun* 11, 1759

5. Zhang, B., Wang, J., Wang, X., Zhu, J., Liu, Q., Shi, Z., Chambers, M. C., Zimmerman, L. J., Shaddox, K. F., Kim, S., Davies, S. R., Wang, S., Wang, P., Kinsinger, C. R., Rivers, R. C., Rodriguez, H., Townsend, R. R., Ellis, M. J., Carr, S. A., Tabb, D. L., Coffey, R. J., Slebos, R. J., Liebler, D. C., and Nci, C. (2014) Proteogenomic characterization of human colon and rectal cancer. *Nature* 513, 382-387

6. Clark, D. J., Dhanasekaran, S. M., Petralia, F., Pan, J., Song, X., Hu, Y., da Veiga Leprevost, F., Reva, B., Lih, T. M., Chang, H. Y., Ma, W., Huang, C., Ricketts, C. J., Chen, L., Krek, A., Li, Y., Rykunov, D., Li, Q. K., Chen, L. S., Ozbek, U., Vasaikar, S., Wu, Y., Yoo, S., Chowdhury, S., Wyczalkowski, M. A., Ji, J., Schnaubelt, M., Kong, A., Sethuraman, S., Avtonomov, D. M., Ao, M., Colaprico, A., Cao, S., Cho, K. C., Kalayci, S., Ma, S., Liu, W., Ruggles, K., Calinawan, A., Gumus, Z. H., Geiszler, D., Kawaler, E., Teo, G. C., Wen, B., Zhang, Y., Keegan, S., Li, K., Chen, F., Edwards, N., Pierorazio, P. M., Chen, X. S., Pavlovich, C. P., Hakimi, A. A., Brominski, G., Hsieh, J. J., Antczak, A., Omelchenko, T., Lubinski, J., Wiznerowicz, M., Linehan, W. M., Kinsinger, C. R., Thiagarajan, M., Boja, E. S., Mesri, M., Hiltke, T., Robles, A. I., Rodriguez, H., Qian, J., Fenyo, D., Zhang, B., Ding, L., Schadt, E., Chinnaiyan, A. M., Zhang, Z., Omenn, G. S., Cieslik, M., Chan, D. W., Nesvizhskii, A. I., Wang, P., Zhang, H., and Clinical Proteomic Tumor Analysis, C. (2019) Integrated Proteogenomic Characterization of Clear Cell Renal Cell Carcinoma. *Cell* 179, 964-983 e931

7. Cao, L., Huang, C., Cui Zhou, D., Hu, Y., Lih, T. M., Savage, S. R., Krug, K., Clark, D. J., Schnaubelt, M., Chen, L., da Veiga Leprevost, F., Eguez, R. V., Yang, W., Pan, J., Wen, B., Dou, Y., Jiang, W., Liao, Y., Shi, Z., Terekhanova, N. V., Cao, S., Lu, R. J., Li, Y., Liu, R., Zhu, H., Ronning, P., Wu, Y., Wyczalkowski, M. A., Easwaran, H., Danilova, L., Mer, A. S., Yoo, S., Wang, J. M., Liu, W., Haibe-Kains, B., Thiagarajan, M., Jewell, S. D., Hostetter, G., Newton, C. J., Li, Q. K., Roehrl, M. H., Fenyo, D., Wang, P., Nesvizhskii, A. I., Mani, D. R., Omenn, G. S., Boja, E. S., Mesri, M., Robles, A. I., Rodriguez, H., Bathe, O. F., Chan, D. W., Hruban, R. H., Ding, L., Zhang, B., Zhang, H., and Clinical Proteomic Tumor Analysis, C. (2021) Proteogenomic characterization of pancreatic ductal adenocarcinoma. *Cell* 184, 5031-5052 e5026

8. Wen, B., Wang, X., and Zhang, B. (2019) PepQuery enables fast, accurate, and convenient proteomic validation of novel genomic alterations. *Genome Res* 29, 485-493
